# The impact of direct‐acting antivirals on hepatitis C viraemia among people who inject drugs in England; real‐world data 2011–2018

**DOI:** 10.1111/jvh.13575

**Published:** 2021-07-29

**Authors:** Megan Bardsley, Ellen Heinsbroek, Ross Harris, Sara Croxford, Claire Edmundson, Vivian Hope, Nasra Hassan, Samreen Ijaz, Sema Mandal, Justin Shute, Sharon J. Hutchinson, Matthew Hickman, Katy Sinka, Emily Phipps

**Affiliations:** ^1^ National Infection Service Public Health England London UK; ^2^ Public Health Institute Liverpool John Moores University Liverpool UK; ^3^ Glasgow Caledonian University Glasgow UK; ^4^ Public Health Scotland Glasgow UK; ^5^ Population Health Sciences Bristol Medical School University of Bristol Bristol UK

**Keywords:** direct‐acting antivirals, elimination, hepatitis C virus, people who inject drugs, treatment as prevention

## Abstract

Direct‐acting antiviral (DAA) therapy for anybody with viraemic HCV infection has been scaled‐up in England since 2017. To assess early impacts, we investigated trends in, and factors associated with, HCV viraemia among people who inject drugs (PWID). We also examined trends in self‐reported treatment access. Bio‐behavioural data from an annual, national surveillance survey of PWID (2011–2018) estimated trends in viraemic prevalence among HCV antibody‐positive PWID. Multivariable logistic regression identified characteristics independently associated with viraemia. Trends in treatment access were examined for PWID with known infection. Between 2011 and 2016, viraemic prevalence among antibody‐positive PWID remained stable (2011, 57.7%; 2016, 55.8%) but decreased in 2017 (49.4%) and 2018 (50.4%) (both *p* < 0.001). After adjustment for demographic and behavioural characteristics, there remained significant reduction in viraemia in 2017 (adjusted odds ratio [aOR] 0.79, 95% CI 0.65–0.94) and 2018 (aOR 0.79, 95% CI 0.66–0.93) compared to 2016. Other factors associated with viraemia were male gender (aOR 1.68, 95% CI 1.53–1.86), geographical region, injecting in past year (aOR 1.26, 95% CI 1.13–1.41), imprisonment (aOR 1.14, 95% CI 1.04–1.31) and homelessness (aOR 1.17, 95% CI 1.04–1.31). Among non‐viraemic PWID with known infection, the proportion reporting ever receiving treatment increased in 2017 (28.7%, *p* < 0.001) and 2018 (38.9%, *p* < 0.001) compared to 2016 (14.5%). In conclusion, there has been a small reduction in HCV viraemia among antibody‐positive PWID in England since 2016, alongside DAA scale‐up, and some indication that treatment access has improved in the same period. Population‐level monitoring and focus on harm reduction is critical for achieving and evaluating elimination.

**Abbreviations**
aOR: Adjusted Odds Ratio
DAA: Direct‐acting antivirals
EPIToPe: Evaluation of the Population Impact of HCV DAA Treatment as prevention for PWID
HBV: Hepatitis B virus
HCV: Hepatitis C virus
MICE: Multiple Imputation by Chained Equations
NIHR: National Institute for Health Protection
OR: Odds Ratio
PWID: People Who Inject Drugs
UAM: Unlinked Anonymous Monitoring
WHO: World Health Organisation

## 1 INTRODUCTION

The global prevalence of hepatitis C virus (HCV) infection is estimated at 1%^1^ and disproportionately affects people who inject drugs (PWID).^2^, ^3^, ^4^ In England, injecting drug use is cited as the risk in over 90% of laboratory reports where a risk factor has been disclosed.^5^ Severe outcomes such as liver cirrhosis and hepatocellular carcinoma contribute to the high economic and health burden of this disease.^6^

The World Health Organisation's (WHO) target to eliminate HCV as a public health threat by 2030 became a possibility in 2011, with the development of highly effective and tolerable direct‐acting antiviral (DAA) therapies.^6^, ^7^, ^8^, ^9^, ^10^ Mathematical modelling has shown that alongside strengthening of harm reduction services, such as needle and syringe programmes (NSP) and opioid substitution therapy (OST), major scale‐up of DAA therapies for PWID is required to meet elimination targets.^11^, ^12^, ^13^, ^14^

In 2015, the English National Health Service (NHS) announced their then single largest investment in new treatment, with a budget of £190 million to fund DAA therapies prioritized for patients with severe liver disease.^15^ The restriction on disease stage was lifted in 2017 and since then treatment is recommended for all those with viraemic HCV infection.^5^, ^16^, ^17^ To drive improvements in access and uptake in local areas, regional operational delivery networks (ODNs) are being utilized to manage HCV treatment decisions and prescribing via a dispersed treatment model.^18^, ^19^ ODNs provide a uniform standard of quality treatment, but work in partnership with healthcare providers and local organizations including primary care services, local authorities and services for PWID to meet local priorities and need.^17^, ^18^, ^19^ A national treatment database has also been developed by NHS England to track HCV treatment uptake and outcomes.^16^, ^17^ Testing for HCV is available in primary care, sexual health clinics and community drug and alcohol services, and more recently has been expanded to community pharmacies.^20^ Solidifying its commitment to these above efforts, in 2018, NHS England set out plans to be the first country in the world to eliminate HCV.^16^

As countries push towards elimination goals, ‘real‐world’ data on the impact of scaling up HCV DAA therapies among PWID are required to track progress or pitfalls. However, few countries are able to measure population‐level changes in HCV viraemia among PWID due to lack of robust data sources.^7^, ^21^ England is one of the few countries in a position to do so, having a long‐standing national surveillance system utilizing an annual cross‐sectional bio‐behavioural survey that monitors blood‐borne viruses and associated risk behaviours among PWID.^22^

We aim to assess the early impact of DAA scale‐up in England by examining changes in HCV viraemia among antibody‐positive PWID between 2011 and 2018. We also examine factors associated with HCV viraemia and assess self‐reported HCV treatment access among those eligible.

## 2 MATERIALS AND METHODS

### 2.1 Data source

#### 2.1.1 The unlinked anonymous monitoring survey of PWID

Data were analysed from the Unlinked Anonymous Monitoring (UAM) Survey of PWID, a national surveillance survey across England, Wales (since 1990) and Northern Ireland (since 2002). The methods of the UAM Survey have been previously described,^23^, ^24^, ^25^ but in brief, PWID recruited through specialist services for people who use drugs are asked to provide a dried blood spot (DBS) sample and self‐complete a demographic and behavioural questionnaire. The questionnaire is linked to the DBS sample, but unlinked from any personal identifying information. Participants are eligible if they have ever injected psychoactive drugs and have not already participated in the same calendar year. Approximately 2,000–3,000 PWID from over 60 sites participate in the UAM Survey each year; recruitment of sites aims to be reflective of the geographical distribution of PWID across England, Wales and Northern Ireland.^26^ The UAM Survey has ethical approval from Public Health England (PHE) and the London Research Ethics Committee (98/2/051).

### 2.2 Laboratory testing

#### 2.2.1 Dried blood spot sample preparation

Dried blood spot samples collected as part of the UAM Survey of PWID are tested for antibodies to HIV (anti‐HIV), hepatitis C (anti‐HCV) and hepatitis B core antibody (anti‐HBc). Presence of antibody indicates a history of infection. For this study, HCV RNA testing to indicate current infection (ie HCV viraemia) was only performed on anti‐HCV‐positive samples. Additional funding from the National Institute for Health Research (NIHR) has facilitated evaluation of the population impact of HCV DAA treatment as prevention for PWID (the EPIToPe project).^27^ EPIToPe^27^ funded historic testing of anti‐HCV‐positive samples collected between 2011 and 2016; testing after 2016 was, and continues to be, funded by PHE. All laboratory testing is carried out at the Virus Reference Department at PHE, Colindale, using previously reported methods.^28^

#### 2.2.2 Lysis and extraction

RNA testing involved elution from the DBS by lysing a 6 mm spot for 2 h at 56°C with 20 µl of proteinase K and 300 µl of ATL lysis buffer (Qiagen products: 19133 and 19076). The entire eluate was extracted on the Qiagen Qiasymphony platform using the Qiasymphony DSP Virus/Pathogen mini kit (Qiagen product: 937036) and ‘cell‐free V6/7 DSP default IC’ protocol. Bacteriophage MS2 was added as the internal control.

#### 2.2.3 Amplification and detection

The qualitative PCR targets the non‐coding region of the HCV genome using the ABI 7500 real‐time thermal cycling with Qiagen TaqMan‐PCR reagents. The multiplex real‐time PCR detects both HCV and MS2 with differently labelled TaqMan probes. Amplification was performed using 20 μl of extract in a 50 μl volume containing 25 μl of QuantiTect Q RT‐PCR mastermix, 3.5 μl of nuclease‐free water, 0.5 μl of QuantiTect RT enzyme and 1 μl of HCV Taqman primer/probe mix (20 pmol of HCV primers (HCV primer 1, HCV primer 2), 5 pmol MS2 primers (MS2 primer 1, MS2 primer 2), 10 pmol of HCV probe and 5 pmol MS2 probe (Applied Biosystems & Metabion)). The primer and probe sequences are provided in Table S1. The reaction mixture was amplified using the following cycling conditions: 50°C for 30 min for the RT step followed by 95°C for 15 min and amplification for 45 cycles at 95°C for 15 s and 60°C for 1 min. Amplification and detection of HCV RNA and MS2 were done using ABI PRISM 7500 Sequence Detection System.

### 2.3 Statistical methods

#### 2.3.1 Inclusion criteria

Participants recruited into the UAM Survey of PWID from survey years 2011–2018 inclusive from England only were included (87.9% of the total sample from across England, Wales and Northern Ireland). Individuals with missing age or gender on the questionnaire were excluded from analyses, as well as HIV‐positive individuals (due to the potential effect of HIV on the anti‐HCV antibody response^29^). Samples that could not be tested for anti‐HCV (because of poor sample quality/insufficient blood volume) were excluded.

#### 2.3.2 Outcome measure

The outcome for the main analyses was viraemic infection, defined as having an anti‐HCV‐positive and an HCV RNA‐positive DBS sample test result. Analyses were conducted only among those anti‐HCV positives.

#### 2.3.3 Classifying pre‐DAA and post‐DAA years

The HCV DAA treatment programme, funded by NHS England specialized commissioning, was introduced in 2014 for compassionate use for patients with end‐stage liver disease, and in 2015 for patients with moderate or severe liver disease (evidence of advanced fibrosis or cirrhosis). DAA treatments were not made widely available to those with milder disease or no fibrosis until 2017 (when restrictions on disease stage were lifted). To reflect this, 2011–2016 were considered to be ‘pre‐DAA’ years and 2017–2018 as the ‘post‐DAA’ years.

#### 2.3.4 Multiple imputation

Multiple imputation by chained equations (MICE) was performed to assign either an RNA‐positive or an RNA‐negative status to anti‐HCV‐positive samples that were insufficient for RNA testing. Missing data were assumed to be missing at random, such that unbiased imputed values could be obtained conditional on observed covariates. Survey year, age, gender, region, history of homeless and imprisonment, country of birth, hepatitis B (HBV) vaccination history and HBV status were used as predictors in the imputation model, and ten imputed data sets were generated. A sensitivity analysis compared the results from MICE to results from non‐imputed data. An additional sensitivity analysis was conducted to include observations for which antibody status was missing (excluded in main analyses) on the basis of the same imputation model.

### 2.4 Statistical analyses

Several demographic and behavioural variables were selected from the UAM Survey of PWID questionnaire and investigated as risk factors for viraemia based on prior literature and hypothesized variables of interest. Age was categorized as binary (<35‐year and ≥35‐year) and geographical region (East of England, London, South East, South West, East Midland, West Midlands, North East, North West and Yorkshire and Humber).

Factors associated with viraemic infection among those anti‐HCV positive were explored using multivariable logistic regression. All variables that had a significant univariable association (Wald *p* < 0.05) with the outcome were added into the multivariable model. Using a backward stepwise approach, a covariate was removed if this (a) did not impact greater than ±10% change in effect estimate of any other variable in the model, and (b) was deemed appropriate by a likelihood ratio test (*p* > 0.05). Variables that were kept in as confounders *a priori* were geographical region, gender, age and injecting in the past year. This modelling approach was performed on complete‐case data, and then, the final model applied to the multiple imputed data set.

To quantify differences in viraemia between regions, a simpler model was fitted comparing the 2017–18 period with 2015–16 and estimated the change in prevalence in each region, whilst controlling for age, sex and other variables included in the main model. Periods were defined in 2‐year blocks rather than individual years to increase power and provide a single estimate of prevalence change over time for each region. A similar approach was used to compare prevalence in 2015–16 and 2017–18 across other subgroups, including age group, gender, injected in last year (yes/no), ever in prison (yes/no) and ever homeless (yes/no). Differences in trends were assessed by the significance of the interaction between the period and subgroup variables.

All analyses were conducted on the multiple imputed data sets using Stata 13 (College Station, TX: StataCorp LP). Summary statistics and proportions were calculated by taking the average across imputed data sets. Statistical tests of differences in proportions and trends over time were conducted using logistic regression, with results combined across imputed data sets to appropriately account for within‐ and between‐imputation variability according to Rubin's rules.

### 2.5 Treatment access

Participants who reported ever testing positive for HCV were asked if they had ever received treatment for their HCV infection; this response was combined with infection status from DBS testing. A cleared infection result (anti‐HCV positive, HCV RNA negative) among people who self‐reported ever receiving treatment was assumed to be reflective of successful access to treatment.

Descriptive trends in the proportion of those with non‐viraemic (cleared) infection who reported ever receiving treatment were presented for 2011–2018. This was not included in analytical modelling due to treatment being on the causal pathway to infection status.

## 3 RESULTS

### 3.1 Characteristics of the study population

There were a total of 20,637 responses from PWID in England who completed a questionnaire and provided a DBS sample between 2011–2018; after excluding those with missing demographics (*n* = 585), those HIV‐positive (*n* = 229) or not tested for anti‐HCV (*n* = 835), a total of 19,039 responses were included from 138 unique study sites (Figure S1).

Among all survey participants included, 73.2% were male and 63.7% were aged 35 or over (Table 1). The North West had the largest number of responses (15.5%) followed by London (14.2%). The majority reported having ever been tested for HCV (84.1%) and having first injected more than 3 years preceding the survey (90.7%). Most respondents had injected in the last year (71.3%). There were high rates of history of imprisonment and homelessness (69.5% and 76.2%, respectively).

**TABLE 1:** Demographic and behavioural characteristics of the study population, including number anti‐HCV positive and HCV RNA positive

Variable	Total sample characteristics (*N* = 19,039)		Number anti‐HCV positive (*N* = 9,650)		Chronic HCV infection (among those anti‐HCV positive) (*N* = 4,761)	
	*n*	% (col)	*n*	% (row)	*n* ^a^	% (row)^b^
**Demographic**						
Year						
2011	2,359	(12.4)	1,054	(44.7)	527	(57.7)
2012	2,807	(14.7)	1,366	(48.7)	694	(57.5)
2013	2,687	(14.1)	1,338	(49.8)	670	(55.6)
2014	2,587	(13.6)	1,296	(50.1)	663	(56.8)
2015	2,240	(11.8)	1,159	(51.7)	562	(55.5)
2016	2,183	(11.5)	1,161	(53.2)	595	(55.8)
2017	2,006	(10.5)	1,077	(53.7)	484	(49.4)
2018	2,170	(11.4)	1,199	(55.3)	566	(50.4)
Gender						
Female	5,106	(26.8)	2,556	(50.1)	1,042	(44.8)
Male	13,933	(73.2)	7,094	(50.9)	3,719	(58.6)
Age (years)						
<35	6,907	(36.3)	2,657	(38.5)	1,339	(54.8)
≥35	12,132	(63.7)	6,993	(57.6)	3,422	(55.0)
Region						
East of England	1,365	(7.2)	585	(42.9)	251	(52.6)
London	2,704	(14.2)	1,595	(59.0)	773	(56.7)
South East	2,200	(11.6)	1,252	(56.9)	648	(55.3)
South West	2,086	(11.0)	982	(47.1)	500	(54.2)
West Midlands	2,026	(10.6)	749	(37.0)	397	(56.0)
North West	2,946	(15.5)	1,903	(64.6)	921	(55.1)
Yorkshire & Humber	2,108	(11.1)	1,104	(52.4)	601	(59.7)
East Midlands	1,960	(10.3)	895	(45.7)	433	(52.0)
North East	1,644	(8.6)	585	(35.6)	237	(46.4)
Birthplace						
Non‐UK	1,219	(6.5)	716	(58.7)	386	(59.0)
UK	17,446	(93.5)	8,742	(50.1)	4,286	(54.6)
**Behavioural**						
Ever had an HCV test						
No	2,912	(15.9)	798	(27.4)	400	(57.1)
Yes	15,412	(84.1)	8,519	(55.3)	4,198	(54.8)
Recent initiate (in past 3 years)						
No	16,729	(90.7)	8,983	(53.7)	4,441	(55.0)
Yes	1,718	(9.3)	424	(24.7)	207	(54.0)
Injected drugs in the past year						
No	5,229	(28.7)	2,303	(44.0)	1,027	(50.6)
Yes	12,986	(71.3)	6,918	(53.3)	3,529	(56.4)
Injected crack in the past month^c^						
No	8,091	(64.0)	3,740	(46.2)	1,818	(54.4)
Yes	4,561	(36.1)	3,012	(66.0)	1,627	(59.1)
Ever been in prison						
No	5,655	(30.5)	2,020	(35.7)	892	(49.2)
Yes	12,870	(69.5)	7,368	(57.3)	3,743	(56.5)
Ever been homeless						
No	4,417	(23.8)	1,751	(39.6)	790	(49.8)
Yes	14,162	(76.2)	7,674	(54.2)	3,868	(56.1)
Ever had transactional sex^d^						
No	15,489	(87.2)	7,674	(49.5)	3,823	(55.6)
Yes	2,281	(12.8)	1,264	(55.4)	572	(50.1)
**Clinical**						
Hepatitis B (ever infected)						
No	16,380	(86.2)	7,474	(45.6)	3,762	(55.3)
Yes	2,633	(13.9)	2,160	(82.0)	991	(53.7)
Had hepatitis B vaccination						
No/not sure	4,903	(26.6)	2,078	(42.4)	1,086	(58.5)
Yes	13,563	(73.5)	7,528	(53.5)	3,527	(53.9)

Note

Missing data in total sample: Birthplace (401), ever had an HCV test (756), recent initiate (610), injected in past year (862), injected crack in past month (334), ever been in prison (552), ever been homeless (495), ever had transactional sex (1,330), HBV infection (26) and HBV vaccination (573).

^a^ The number with chronic infection excludes samples missing RNA status.

^b^ The denominator for chronic infection percentages includes samples missing RNA that had data imputed with multiple‐imputation by chain equations (MICE).

^c^ Among those who injected (any drug) in the past year.

^d^ Transactional sex is defined as ever receiving money, goods or drugs in exchange for sex.

**FIGURE 1:**
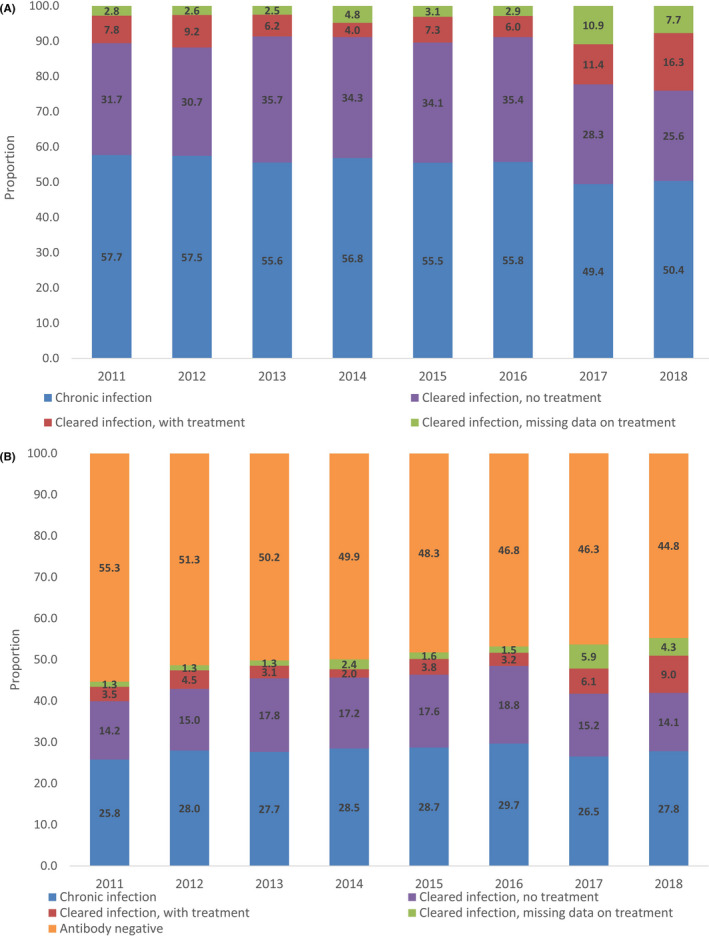
Prevalence of chronic and cleared HCV infection among PWID in England, 2011–2018. Figures present (A) HCV antibody positives only and (B) all respondents. HCV, hepatitis C infection; PWID, People who inject drugs. Antibody‐positive samples that were missing RNA had data imputed with MICE

A total of 9,650 samples (50.7%) had an anti‐HCV‐positive test result and were included in the main analysis. Unadjusted anti‐HCV prevalence increased each year between 2011 and 2018 (test for trend *p* < 0.001), rising from 44.7% to 55.3%, respectively (Table 1, Figure 1A). The exclusion of samples not tested for anti‐HCV had no impact on these results (Table S2a) or on subsequent multivariable results (Table S2b).

### 3.2 Factors associated with HCV viraemia

In the adjusted model, HCV viraemia among those anti‐HCV positive was associated with survey year (see ‘trends in HCV viraemia’ section), male gender (adjusted odds ratio [aOR] 1.68, 95% confidence interval [CI] 1.53–1.86), region (Yorkshire and Humber: aOR 1.29, 95% CI 1.04–1.60; North East: aOR 0.72, 95% CI 0.56–0.92; both compared to East of England), having injected in the past year (aOR 1.26, 95% CI 1.13–1.41), history of imprisonment (aOR 1.14, 95% CI 1.01–1.29) and history of homelessness (aOR 1.17, 95% CI 1.04–1.31) (Table 2).

**TABLE 2:** Odds of chronic HCV infection among antibody‐positive PWID in England; results from logistic regression with MICE

Variable	Univariable					Multivariable				
	OR	95% CI			*p*‐value	aOR	95% CI			*p*‐value
**Demographic**										
Year										
2011	1.08	0.90	–	1.30	0.39	1.04	0.87	–	1.25	0.80
2012	1.07	0.90	–	1.27	0.42	1.04	0.87	–	1.24	0.66
2013	0.99	0.84	–	1.17	0.92	0.99	0.84	–	1.16	0.87
2014	1.04	0.88	–	1.24	0.62	1.02	0.86	–	1.22	0.79
2015	0.99	0.83	–	1.17	0.91	0.98	0.83	–	1.17	0.83
2016	–		–		–	–		–		–
2017	0.78	0.65	–	0.93	0.01	0.79	0.65	‐	0.94	0.01
2018	0.80	0.68	–	0.95	0.01	0.78	0.66	‐	0.93	0.01
Gender										
Female	–		–		–	–		–		–
Male	1.74	1.58	–	1.91	0.00	1.68	1.53	–	1.86	0.00
Age (years)										
<35	–		–		–	–		–		–
≥35	1.01	0.92	–	1.11	0.89	0.96	0.86		1.06	0.41
Region										
East of England	–		–		–	–		–		–
London	1.18	0.97	–	1.45	0.10	1.22	0.99	–	1.50	0.06
South East	1.11	0.90	–	1.38	0.32	1.07	0.86	–	1.33	0.53
South West	1.07	0.86	–	1.33	0.55	1.00	0.80	–	1.24	0.97
West Midlands	1.15	0.91	–	1.44	0.23	1.11	0.88	–	1.39	0.40
North West	1.11	0.91	–	1.35	0.32	1.09	0.89	–	1.33	0.41
Yorkshire & Humber	1.33	1.08	–	1.65	0.01	1.29	1.04	–	1.60	0.02
East Midlands	0.98	0.79	–	1.21	0.83	0.89	0.71	–	1.10	0.28
North East	0.78	0.61	–	1.00	0.05	0.72	0.56	–	0.92	0.01
Birthplace										
Non‐UK	–		–		–					
UK	0.83	0.71		0.98	0.03					
**Behavioural**										
Injected drugs in the past year										
No	–		–		–	–		–		–
Yes	1.26	1.14	–	1.39	0.00	1.26	1.13	–	1.41	0.00
Injected crack in the past month^a^										
No	–		–		–					
Yes	1.22	1.10	–	1.34	0.00					
Ever been in prison										
No	–		–		–	–		–		–
Yes	1.34	1.20	–	1.50	0.00	1.14	1.01	–	1.29	0.03
Ever been homeless										
No	–		–		–	–		–		–
Yes	1.29	1.15	–	1.44	0.00	1.17	1.04	–	1.31	0.01
Recent initiate (in past 3 years)										
No	–		–		–					
Yes	0.96	0.78		1.17	0.68					
Ever had transactional sex^b^										
No	–		–		–					
Yes	0.80	0.71		0.91	0.00					

Abbreviations: aOR, adjusted Odds Ratio; CI, Confidence Interval.

^a^ Among those who injected (any drug) in the past year. Antibody‐positive samples that were missing RNA had data imputed with MICE.

^b^ Transactional sex is defined as ever receiving money, goods or drugs in exchange for sex.

Of the anti‐HCV‐positive samples available for 2011–2018, *n* = 1,167 (6.1%) were insufficient for HCV RNA testing and had an RNA result imputed. A sensitivity analysis comparing these findings to a multivariable model without imputation showed no differences in significant associations (Table S3).

### 3.3 Trends in HCV viraemia

Between 2011 and 2016, the prevalence of viraemia among antibody‐positive PWID in England remained stable, at around 56.5% (*p* = 0.275) (Table 1, Figure 1B). The prevalence of viraemic infection among antibody positives then fell in 2017 to 49.4% (*p* = 0.006) and then remained similar (*p* = 0.68) in 2018 at 50.4%.

After adjustment for demographic and behavioural characteristics, respondents in 2017 and 2018 had significantly lower odds of viraemia compared with those in 2016 (2017: aOR 0.79, 95% CI 0.65–0.94, 2018: aOR 0.78 95% CI 0.66–0.93) (Table 2).

**FIGURE 2:**
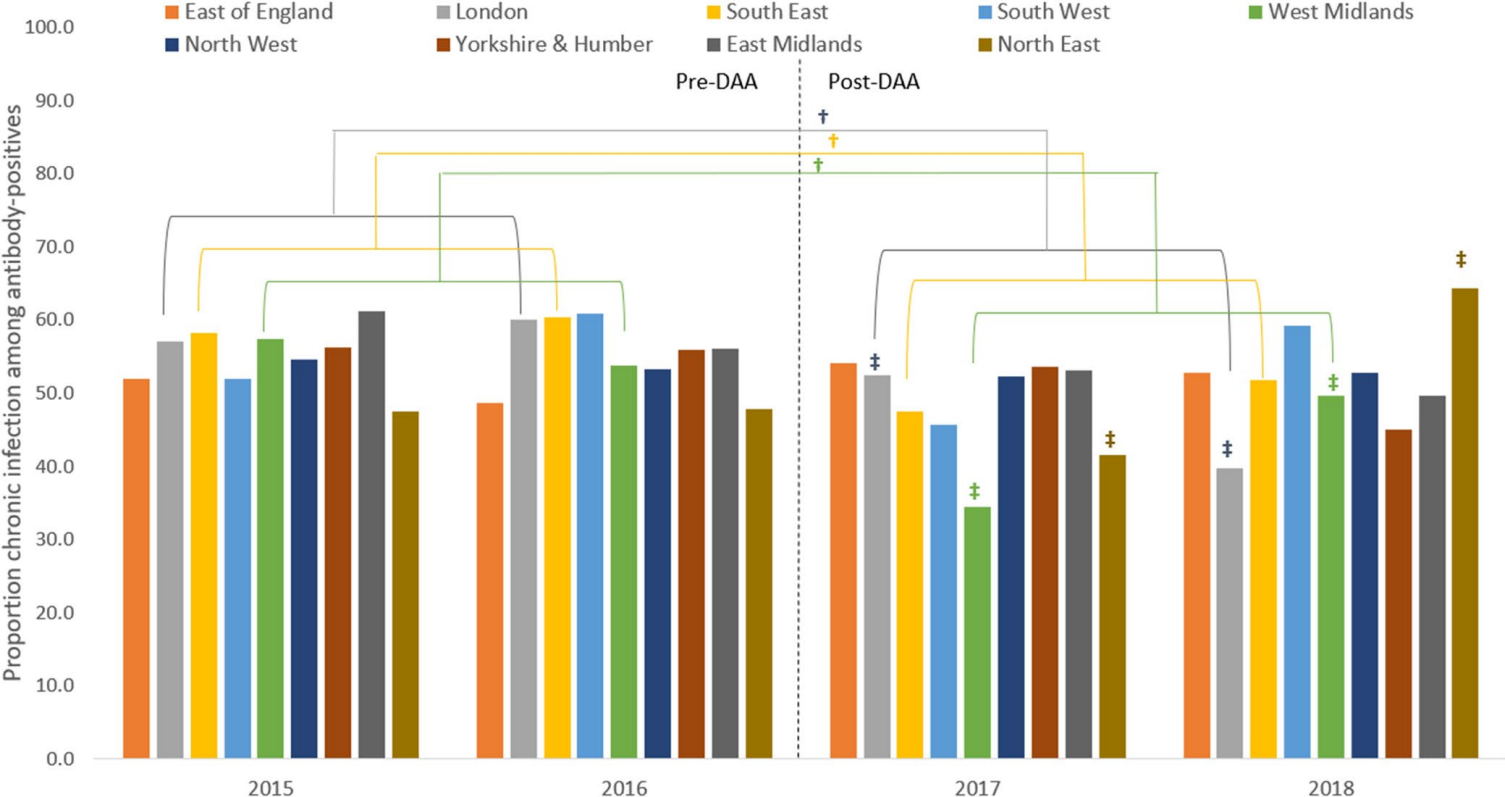
Prevalence of chronic HCV infection among antibody‐positive PWID in England, 2015 to 2018, by region. HCV, hepatitis C infection; PWID, People who inject drugs. †Regions with significant difference between 2017/18 and 2015/16: London, South East, West Midlands. ‡Regions with significant within‐period variability for 2017/18: London, West Midlands, North East. No regions had within‐period variability for 2015/16. Antibody‐positive samples that were missing RNA had data imputed with MICE

Trends in the proportion with viraemic infection differed between regions. There was evidence for differences in viraemia in 2017–18 compared to 2015–16 in London, South East and West Midlands, with all three having lower odds of viraemia in 2017–18 compared to 2015–16 (Table 3, Figure 2). There was some within‐period variability in 2017–18 for London, West Midlands and the North East (Figure 2). We found no evidence of differences in trends according to other subgroups (gender, age, injecting in past year, history of imprisonment and homelessness, minimum *p*‐value = 0.161, data not shown).

**TABLE 3:** Odds of chronic HCV infection among antibody‐positive PWID by region in 2015/16 and 2017/18; results from logistic regression with MICE

Region	2017/2018 (compared to 2015/16)				
	aOR	95% CI			*p*‐value
East of England	**1.19**	0.75	–	1.88	0.46
London	**0.57**	0.41	–	0.80	0.00
South East	**0.68**	0.49	–	0.95	0.02
South West	**0.98**	0.68	–	1.43	0.94
West Midlands	**0.61**	0.39	–	0.98	0.04
North West	**0.93**	0.70	–	1.23	0.60
Yorkshire & Humber	**0.80**	0.54	–	1.19	0.27
East Midlands	**0.79**	0.51	–	1.20	0.26
North East	**1.10**	0.62	–	1.92	0.75

Note

Adjusted for gender, age, injecting in past year, history of imprisonment and homelessness.

Antibody‐positive samples that were missing RNA had data imputed with MICE.

### 3.4 HCV treatment uptake

Among those with cleared infection and for whom treatment evidence was available, the proportion of participants who reported ever receiving HCV treatment increased in the post‐DAA era (2017: 28.7%, OR 1.39, 95% CI 1.15–1.67; 2018: 38.9%, OR 1.59, 95% CI 1.32–1.91; both compared to 2016) (Table 4).

**TABLE 4:** Estimates of treatment‐induced viral clearance among individuals with cleared HCV infection who were not missing data on self‐reported treatment history

Year	Number with cleared HCV and data on treatment history	Proportion ever receiving HCV treatment	Odds of receiving HCV treatment	95% CI			P‐value
2011	4,164	19.7%	1.18	0.98	–	1.42	0.08
2012	5,455	23.1%	1.24	1.04	–	1.47	0.02
2013	5,610	14.8%	0.98	0.83	–	1.16	0.86
2014	4,971	10.5%	1.05	0.88	–	1.25	0.60
2015	4,800	17.7%	1.06	0.89	–	1.26	0.53
2016 (base)	4,804	14.5%	1.00		–		–
2017	4,274	28.7%	1.39	1.15	–	1.67	0.00
2018	5,026	38.9%	1.59	1.32	–	1.91	0.00

## 4 DISCUSSION

These analyses of national bio‐behavioural surveillance data show that there has been a slight decline in the prevalence of viraemic HCV infection among ever‐infected PWID, associated with a substantial increase in HCV treatment uptake in England, since the scale‐up of DAA treatments provided through the NHS. Participants who have evidence of viraemic HCV infection tended to be male, recent injectors (in past year) and have a history of homelessness or incarceration. We found some evidence of regional variability in trends in HCV viraemia, although this must be interpreted with caution.

There have been only two other studies to use national surveillance data to examine the population‐level impact of HCV treatment scale‐up among PWID. In Scotland, recent findings from a similar bio‐behavioural survey demonstrate that rapid scale‐up of DAAs in 2017 through community drug services succeeded in increasing treatment uptake and reducing viraemic prevalence in the Tayside ‘intervention’ region more so than the rest of Scotland. HCV viraemia among PWID ever infected in Scotland fell from 67% in 2015–16 to 55% in 2017–18, but this was more pronounced in Tayside (58% to 44%) compared to the rest of Scotland (65%–55%). In the same time, treatment uptake (ever) in Scotland increased from 17%–38% (35%–65% in Tayside and 17%–40% in the rest of Scotland). Whist it is difficult to directly compare English and Scottish results—namely because England has no defined ‘intervention’ site and we primarily present data at the national level—both Scotland and England observed a similar downward trend in HCV viraemic prevalence after DAA scale‐up. However, English data are reflective of the impact of at least two full years post‐DAA roll‐out, whereas data collected from Scotland (in 2017–18) reflect approximately 1 year of DAA scale‐up (which occurred from 2017). It is relevant to note here that the choice of 2016 as the baseline year in our analyses was taken to reflect when DAAs became more widely available. However, in practice, a small subset of patients were treated from as early as 2014 when DAAs were first commissioned by NHS England.^30^ It is possible, therefore, that we have underestimated the decline in viraemia in PWID in England's ‘post‐DAA era’. Australia has also measured the population impact of DAA scale‐up where unrestricted access to DAA was introduced in 2016; between 2015 and 2017, treatment uptake (ever) among Australian PWID quadrupled (from 10% to 41%), and viraemic prevalence almost halved (from 43% to 25%^7^).

It is encouraging that increased treatment provision with DAA therapies (+131% in tax year 2018 to 2019 compared to pre‐2015 levels^5^) are beginning to make an impact on reducing the burden of HCV in England. Our finding of a reduction in HCV viraemia is corroborated by NHS Blood and Transplant Service data showing the number of liver transplant registrations and operations undertaken for HCV‐associated cirrhosis and hepatocellular carcinoma fell by 44% and 29% in 2018, respectively, when compared to pre‐2015 levels,^5^ and this is evidence of early impact of DAAs on transplants was also observed in a data linkage study of HCV diagnosed individuals.^31^ There has also been a 20% fall in HCV‐related deaths in England between 2015 and 2018, exceeding the WHO target 3 years early and twofold.^6^, ^32^ However, these are only early signs of improvement, and there is evidence to suggest the number of new infections may have risen in 2018.^5^ Our study shows that the number of PWIDs ever exposed to HCV infection continues to rise, indicative that it may be effective treatment, more than successful prevention that is currently controlling HCV viraemia in the PWID population.

Unless the coverage and intensity of primary prevention interventions increases, there will be re‐infections after successful DAA treatment.^33^ A multifaceted approach including harm reduction, testing and linkage to care in addition to treatment is required, and there is much progress to be made; the current proportion of PWID reporting adequate needle and syringe provision in England, Wales and Northern Ireland is suboptimal (64% of respondents in the 2018 UAM Survey^23^) and this is especially concerning given the lack of improvement in and high levels of reported sharing of injecting paraphernalia (39% of current PWID reported sharing equipment in 2018^23^). Our study demonstrates that the majority of PWID with past infection did not receive treatment for their HCV, even as recently as 2018. Specific commitments to strengthen and expand NSP, OST and other harm reduction services are needed. Moreover, a move towards ‘non‐traditional’ prevention pathways that involve peer‐support mechanisms will be required to reach PWID who are less likely to seek out health care and to ensure they are supported along the full care pathway. Innovations such as outreach treatment models have previously demonstrated the power of decentralization of care into community‐based settings.^34^, ^35^, ^36^, ^37^

Our findings from multivariable analyses showing that viraemic infection is often associated with history of homelessness and incarceration are consistent with previous literature.^2^, ^23^, ^28^, ^38^, ^39^, ^40^, ^41^ Being exposed to these risks could be significant drivers of transmission, and there is an opportunity for individual and public health benefits to target individuals in these underserved and marginalized communities. Prison‐ and shelter‐based harm reduction interventions (including OST and NSP), effective linkage to prevention and treatment services before, during and after prison release, and community‐based peer workers have been shown to reduce injecting risk, increase treatment uptake and do not result in increased drug use.^39^, ^41^, ^42^ However, the current indication is that improvements to implementation of these interventions are needed in order for prison and shelter settings in England to be able to adequately support HCV elimination.^39^, ^40^, ^42^, ^43^ Changes to funding structures, peer‐support systems and policies are required in order to reduce the burden of HCV infection among PWID in these settings.

The data source for this study, a repeated, cross‐sectional and bio‐behavioural survey of PWID, is one of only four of its kind globally.^7^, ^21^, ^44^ The UAM Survey is nationally representative,^26^ and the data used in this analysis utilized large annual samples of over 2,000 PWID. However, we acknowledge several limitations. Firstly, there will be sampling variability between years, which could explain some differences in HCV infection status between the survey years. The region‐specific results in particular may be affected by changes in recruitment sites over time and sampling variability; the results presented here should not be interpreted as treatment ‘working’ or not in particular areas. Elimination initiatives are locally determined and driven by epidemiological intelligence, priorities, clinical capacity and funding; there is no centralized and publicly available summary of regional projects. Variation can be reasonably assumed to be intrinsic to this elimination approach in England and is likely to reflect a range of factors, including urban and rural difference (eg East of England predominantly rural region whilst London is almost all urban) and local and regional variations in service delivery and innovation (eg Find and Treat Van in London delivering HCV testing and treatment in this region only). Whilst there was no evidence that trends differed according to age, sex, recent injecting behaviour, homelessness and imprisonment, there is little power to detect differences in what is, so far, a modest trend.

Secondly, the behavioural data are self‐reported and therefore may be subject to social desirability and recall bias. Moreover, detailed information on injecting risk behaviours was not available, as these data are only collected for those who had injected during the month prior to participation, which limits interpretation and testing for differential intervention effects by intensity of injecting risk. Thirdly, the data on HCV treatment uptake are self‐reported and not validated, and represent ever receiving treatment, which should be interpreted with caution, as it reflects perception of care at any point during the individual's HCV infection. We cannot discriminate whether DAA or other therapies were given, although the UAM survey has been modified in 2020 to capture more specific data on HCV treatment. We also cannot comment on reinfection after successful HCV treatment as this information is not currently directly captured through the UAM Survey, although there are plans to collect this in future. Fourthly, the eligibility criteria of the UAM Survey prevent people participating multiple times in 1 year, but due to its anonymous nature those people who participate in more than 1 year cannot be linked over time. Finally, it is possible that antibody and viraemic prevalence in this study is not representative of the general population of PWID due to the sampling method; respondents are only those already in contact with specialist drug or alcohol services.^45^ We also excluded a small number of HIV‐positive people from analyses, as HIV and HCV‐coinfected people may remain HCV‐seronegative.^29^ Whilst numbers excluded each year were consistently small, it is possible that HCV prevalence and self‐reported treatment may be different among this excluded group compared to people who are HIV‐negative.

In conclusion, our study adds to the scarce availability of ‘real‐world’ evidence demonstrating that scaling up DAA treatments can lead to reduced HCV viraemic prevalence among PWID. We show treatment uptake has improved in England in recent years but is still suboptimal. There are many challenges that lie ahead for HCV elimination, and whilst revolutionary, DAA treatment is only one element of a comprehensive elimination package. Radical changes to support collaborative work on prevention, testing and linkage to care among PWID is necessary, which will only become more important in light of the impact of the COVID‐19 pandemic and its associated restrictions that have negative impacts on service provision and access to harm reduction, testing and treatment.^46^, ^47^ Future rounds of the UAM Survey will be critical to be able to evaluate our continued progress towards HCV elimination targets.

## CONFLICT OF INTEREST

None to declare.

## AUTHOR CONTRIBUTIONS

The UAM Survey was led by EH, SC, KS and EP and was implemented by EH, SC, KS, EP, CE and MB over the study period. VH had historical involvement with overseeing the Survey and provided topic area expertise. SI, JS and NH performed testing on dried blood spot samples. MH, SH, SM, VH and EH conceived and designed the EPIToPe study which this study contributes to. MB, EH and RS conceived the statistical analysis plan and MB performed the analyses and generation of result tables and figures with support from RH. MB drafted the manuscript, and all authors fed into the review process to develop the final manuscript. We also acknowledge support from NIHR Health Protection Research Unit (HPRU) in Behavioural Science and Evaluation and HPRU in Blood Borne and Sexually Transmitted Infections.

## Supporting information

App S1

## Data Availability

The data that support the findings of this study are available on request from the corresponding author. The data are not publicly available due to privacy or ethical restrictions.

## References

[1] The Polaris Observatory HCV Collaborators . Global prevalence and genotype distribution of hepatitis C virus infection in 2015: a modelling study. Lancet Gastroenterol Hepatol. 2017;2(3):161‐176. (Electronic). 28404132 10.1016/S2468-1253(16)30181-9

[2] Nelson PK , Mathers BM , Cowie B , et al. Global epidemiology of hepatitis B and hepatitis C in people who inject drugs: results of systematic reviews. Lancet. 2011;378(9791):571‐583. 21802134 10.1016/S0140-6736(11)61097-0 PMC3285467

[3] Degenhardt L , Peacock A , Colledge S , et al. Global prevalence of injecting drug use and sociodemographic characteristics and prevalence of HIV, HBV, and HCV in people who inject drugs: a multistage systematic review. Lancet Global Health. 2017;5(12):e1192‐e1207. 29074409 10.1016/S2214-109X(17)30375-3 PMC5683738

[4] Wiessing L , Ferri M , Grady B , et al. Hepatitis C virus infection epidemiology among people who inject drugs in Europe: a systematic review of data for scaling up treatment and prevention. PLoS ONE. 2014;9(7):1932‐6203 (Electronic). 10.1371/journal.pone.0103345 PMC4113410 25068274

[5] Harris HE , Edmundson C , Costella A , Harris R , Mandal S , Contributors a . Hepatitis C in England, 2020 report: Working to eliminate hepatitis C as a major public health treat. London: Public Health England; May 2020.

[6] World Health Organisation . Global Health Sector Strategy on Viral Hepatitis 2016–2021: towards ending viral hepatitis. Geneva, Switzerland: World Health Organisation; 2016.

[7] Iversen J , Dore GJ , Catlett B , Cunningham P , Grebely J , Maher L . Association between rapid utilisation of direct hepatitis C antivirals and decline in the prevalence of viremia among people who inject drugs in Australia. J Hepatol. 2019;70(1):33‐39. 30367897 10.1016/j.jhep.2018.09.030

[8] Harris RJ , Martin NK , Rand E , et al. New treatments for hepatitis C virus (HCV): scope for preventing liver disease and HCV transmission in England. Journal of Viral Hepatitis. 2016;23:1365‐2893 (Electronic). 10.1111/jvh.12529 PMC4982023 27025238

[9] Jacobson IM , McHutchison JG , Dusheiko G , et al. Telaprevir for previously untreated chronic hepatitis C virus infection. N Engl J Med. 2011;364:1533‐4406 (Electronic). 21696307 10.1056/NEJMoa1012912

[10] World Health Organisation . Guidelines for the screening, care and treatment of persons with chronic hepatitis C infection. Geneva, Switzerland: World Health Organisation; 2016.

[11] Innes H , Goldberg D , Dillon J , Hutchinson SJ . Strategies for the treatment of Hepatitis C in an era of interferon‐free therapies: what public health outcomes do we value most? Gut. 2015;64:1468‐3288 (Electronic). 10.1136/gutjnl-2014-308166 25378522

[12] Martin NK , Hickman M , Hutchinson SJ , Goldberg DJ , Vickerman P . Combination interventions to prevent HCV transmission among people who inject drugs: modeling the impact of antiviral treatment, needle and syringe programs, and opiate substitution therapy. Clin Infect Dis. 2013;57(suppl 2):S39‐S45. 23884064 10.1093/cid/cit296 PMC3722076

[13] Vickerman P , Martin N , Turner K , Hickman M . Can needle and syringe programmes and opiate substitution therapy achieve substantial reductions in hepatitis C virus prevalence? Model projections for different epidemic settings. Addiction. 2012;107:1360‐1443 (Electronic). 22564041 10.1111/j.1360-0443.2012.03932.x

[14] Fraser H , Martin NK , Brummer‐Korvenkontio H , et al. Model projections on the impact of HCV treatment in the prevention of HCV transmission among people who inject drugs in Europe. J Hepatol. 2018;68(3):402‐411. 29080808 10.1016/j.jhep.2017.10.010 PMC5841161

[15] NHS England Thousands more patients to be cured of hepatitis C. 2015. http://www.england.nhs.uk/2015/06/10/patients‐hep‐c/. Accessed January 1, 2021.

[16] NHS England NHS England sets out plans to be first in the world to eliminate Hepatitis C. 2018. https://www.england.nhs.uk/2018/01/hepatitis‐c‐2/. Accessed January 1, 2021.

[17] NHS England . Service Specifications‐ Operational Delivery Networks for Hepatitis C Care in Adults. NHS England; 2015.

[18] NHS England BI1: Improving HCV Treatment Pathways through ODNs. 2016. https://www.england.nhs.uk/publication/bi1‐improving‐hcv‐treatment‐pathways‐through‐odns/. Accessed January 1, 2021.

[19] NHS England Operational Delivery Networks. https://www.england.nhs.uk/ourwork/part‐rel/odn/. Accessed January 1, 2021.

[20] Public Health England . Hepatitis C in the UK 2020; Working to eliminate hepatitis C as a major public health threat. London: Public Health England; 2020.

[21] Palmateer NE , McAuley A , Dillon JF , et al. Reduction in the population prevalence of HCV viraemia among people who inject drugs associated with scale‐up of direct‐acting antiviral therapy in community drug services: real world data. Addiction. 2021. [Epub ahead of print]. 10.1111/add.15459 33651446

[22] Public Health England People who inject drugs: HIV and viral hepatitis monitoring. https://www.gov.uk/government/publications/people‐who‐inject‐drugs‐hiv‐and‐viral‐hepatitis‐monitoring. Accessed January 1, 2021.

[23] Public Health England . Unlinked Anonymous Monitoring (UAM) Survey of HIV and viral hepatitis among PWID: 2019 report2019.

[24] Noone A , Durante AJ , Brady AR , et al. HIV infection in injecting drug users attending centres in England and Wales, 1990–1991. AIDS. 1993;7(11):1501‐1507. 8280418 10.1097/00002030-199311000-00015

[25] Hope VD , Harris RJ , De Angelis D , et al. Two decades of successes and failures in controlling the transmission of HIV through injecting drug use in England and Wales, 1990 to 2011. Eurosurveillance. 2014;19(14):20762. 24739984 10.2807/1560-7917.es2014.19.14.20762

[26] The unlinked anonymous HIV prevalence monitoring programme in England and Wales: preliminary results. (0144‐1108 (Print)). 1669780

[27] Hickman M , Dillon JF , Elliott L , et al. Evaluating the population impact of hepatitis C direct acting antiviral treatment as prevention for people who inject drugs (EPIToPe) ‐ a natural experiment (protocol). BMJ Open. 2019;9(9):e029538. 10.1136/bmjopen-2019-029538 PMC6773339 31551376

[28] Cullen KJ , Hope VD , Croxford S , Shute J , Ncube F , Parry JV . Factors associated with recently acquired hepatitis C virus infection in people who inject drugs in England, Wales and Northern Ireland: new findings from an unlinked anonymous monitoring survey. Epidemiol Infect. 2015;143(7):1469‐4409. 10.1017/S0950268814002040 PMC9507190 25119383

[29] Juniastuti , Utsumi Takako , Nasronudin , et al. High rate of seronegative HCV infection in HIV‐positive patients. Biomed Rep. 2014;2(1):79‐84. 24649073 10.3892/br.2013.188 PMC3917057

[30] Harris HE , Costella A , Mandal S & Contributors. a Hepatitis C treatment monitoring in England: Content, completeness and preliminary findings from the Hepatitis C patient registry and treatment outcome system: Public Health England; November 2018 .

[31] Ireland G , Simmons R , Hickman M , Ramsay M , Sabin C , Mandal S . Monitoring liver transplant rates in persons diagnosed with hepatitis C: a data linkage study, England 2008 to 2017. Eurosurveillance. 2019;24(1900176). 10.2807/1560-7917.ES.2019.24.41.1900176 PMC6794990 31615597

[32] Office for National Statistics. Deaths: ONS 2020. https://www.ons.gov.uk/peoplepopulationandcommunity/birthsdeathsandmarriages/deaths. Accessed January 1, 2021.

[33] Asher AK , Portillo CJ , Cooper BA , Dawson‐Rose C , Vlahov D , Page KA . Clinicians' views of hepatitis c virus treatment candidacy with direct‐acting antiviral regimens for people who inject drugs. Subst Use Misuse. 2016;51(9):1532–2491 (Electronic). 10.3109/10826084.2016.1161054 PMC6907073 27219274

[34] Hepatitis C Trust The Hepatitis C Trust Outreach & Testing Van; 2011. http://www.hcvaction.org.uk/resource/hepatitis‐c‐trust‐outreach‐testing‐van. Accessed January 1, 2021.

[35] Kings Health Partners Transforming hepatitis C treatment from cure to elimination. 2018. https://www.kingshealthpartners.org/latest/1884‐transforming‐hepatitis‐c‐treatment‐from‐cure‐to‐elimination. Accessed January 1, 2021.

[36] Alcorn K London: hepatitis C elimination depends on testing scale‐up. 2020. http://www.infohep.org/page/3548419/. Accessed January 1, 2021.

[37] Abbvie For hepatitis C, seeking the end of the road in Washington state. 2019. https://stories.abbvie.com/stories/for‐hepatitis‐c‐seeking‐end‐road‐in‐washington‐state.htm. Accessed January 1, 2021.

[38] Stone J , Fraser H , Lim AG , et al. Incarceration history and risk of HIV and hepatitis C virus acquisition among people who inject drugs: a systematic review and meta‐analysis. Lancet Infect Dis. 2018;18(12):1397‐1409. 30385157 10.1016/S1473-3099(18)30469-9 PMC6280039

[39] Perrett SE , Plimmer A , Shankar AG , Craine N . Prevalence of HCV in prisons in Wales, UK and the impact of moving to opt‐out HCV testing. J Public Health. 2020;42:1741‐3850 (Electronic). 10.1093/pubmed/fdaa022 32090269

[40] Arain A , Robaeys G , Stöver H . Hepatitis C in European prisons: a call for an evidence‐informed response. BMC Infect Dis. 2014;14:1471‐2334 (Electronic). 10.1186/1471-2334-14-S6-S17 PMC4178549 25252822

[41] Aisyah DN , Shallcross L , Hayward A , et al. Hepatitis C among vulnerable populations: a seroprevalence study of homeless, people who inject drugs and prisoners in London. J Viral Hepat. 2018;25(11):1365‐2893. (Electronic). 10.1111/jvh.12936 29851232

[42] Crowley D , Murtagh R , Cullen W , et al. Evaluating peer‐supported screening as a hepatitis C case‐finding model in prisoners. Harm Reduct J. 2019;16(1):1477‐7517 (Electronic). 10.1186/s12954-019-0313-7 PMC6612120 31277665

[43] Jack K , Thomson B , Irving W . Testing for hepatitis C virus infection in UK prisons: What actually happens? J Viral Hepat. 2019;26:1365‐2893 (Electronic). 10.1111/jvh.13071 30702194

[44] Tarasuk J , Ogunnaike‐Cooke S , Archibald C , et al. Key findings from a national enhanced HIV surveillance system: 2010 ‐ 2012. Can Commun Dis Rep. 2014;40:1188‐4169 (Print). 10.14745/ccdr.v40i18a05 PMC5933856 29769871

[45] Hope VD , Hickman M , Ngui SL , et al. Measuring the incidence, prevalence and genetic relatedness of hepatitis C infections among a community recruited sample of injecting drug users, using dried blood spots. J Viral Hepat. 2011;18(4):262‐270. 20456636 10.1111/j.1365-2893.2010.01297.x

[46] Whitfield M , Reed H , Webster J , Hope V . The impact of COVID‐19 restrictions on needle and syringe programme provision and coverage in England. Int J Drug Policy. 2020;83:102851. 32736959 10.1016/j.drugpo.2020.102851 PMC7362866

[47] Picchio CA , Valencia J , Doran J , et al. The impact of the COVID‐19 pandemic on harm reduction services in Spain. Harm Reduction Journal. 2020;17(1):87. 33143699 10.1186/s12954-020-00432-w PMC7609370

