# Supplementary material for: The impact of direct‐acting antivirals on hepatitis C viraemia among people who inject drugs in England; real‐world data 2011–2018
Source: J Viral Hepat. 2021 Jul 29;28(10):1452–63. doi: 10.1111/jvh.13575 (PMC9290701; doi:10.1111/jvh.13575)
Supplement: Supplementary file 1 — App S1 [file JVH-28-1452-s001.docx]

# Appendix

## Appendix figure 1

### Study flow diagram. DBS= Dried Blood Spot

##
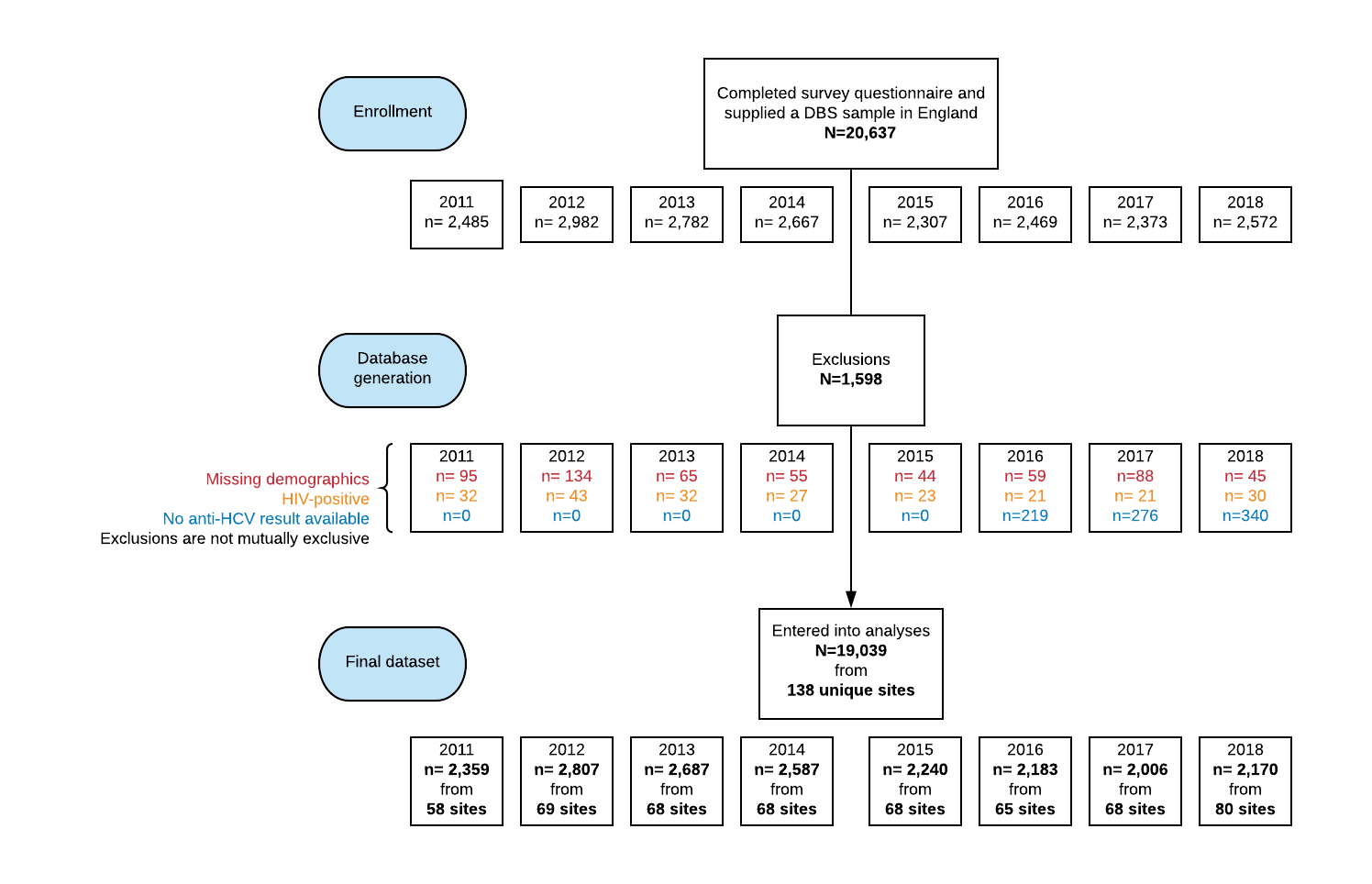


## Appendix table 1

**Primers used for in-house HCV RT-PCR**

| **Primer name** | **Primer sequences** |
| --- | --- |
| HCV Primer 1 | 5'- GTCTAGCCATGGCGTTAGTA -3' |
| HCV Primer 2 | 5'- GTACTCACCGGTTCCGC -3' |
| HCV Probe | 5'FAM- CCCTCCCGGGAGAGCCATAGTG-3'TAMRA |
| MS2 Primer 1 | 5’- TGGCACTACCCCTCTCCGTATTCACG-3’ |
| MS2 Primer 2 | 5’- GTACGGGCGACCCCACGATGAC-3’ |
| MS2 Probe | 5’VIC- CACATCGATAGATCAAGGTGCCTACAAGC -3'TAMRA' |

## Appendix table 2

**Table 2a: Sensitivity analysis; odds of chronic HCV infection among antibody-positive PWID in England, comparing main model (samples missing anti-HCV excluded) with alternate model (samples missing anti-HCV imputed with MICE)**

|  |  | **Imputed model (incl. missing anti-HCV)-proportion anti-HCV positive** | **Main model (excl. missing anti-HCV)-proportion anti-HCV positive** |
| --- | --- | --- | --- |
|  |  |  |  |
| **Year** | 2011 | 44.7% | 44.7% |
|  | 2012 | 48.7% | 48.7% |
|  | 2013 | 49.8% | 49.8% |
|  | 2014 | 50.1% | 50.1% |
|  | 2015 | 51.7% | 51.7% |
|  | 2016 | 53.5% | 53.2% |
|  | 2017 | 54.1% | 53.7% |
|  | 2018 | 55.4% | 55.3% |

**Table 2b: Sensitivity analysis; anti-HCV prevalence between 2011 and 2018, comparing main model (samples missing anti-HCV excluded) with alternate model (samples missing anti-HCV imputed with MICE)**

|  |  | **Imputed model (including missing anti-HCV) multivariable results** | | | | | **Main model (excluding missing anti-HCV) multivariable results** | | | | |
| --- | --- | --- | --- | --- | --- | --- | --- | --- | --- | --- | --- |
|  |  |  |  |  |  |  |  |  |  |  |  |
| **Variable** |  | **aOR** | **95% CI** | | | **p-value** | **aOR** | **95% CI** | | | **p-value** |
| ***Demographic*** |  |  |  |  |  |  |  |  |  |  |  |
| **Year** | 2011 | **1.05** | 0.86 | - | 1.28 | 0.64 | **1.04** | 0.87 | - | 1.25 | 0.80 |
|  | 2012 | **1.04** | 0.87 | - | 1.24 | 0.69 | **1.04** | 0.87 | - | 1.24 | 0.66 |
|  | 2013 | **0.99** | 0.83 | - | 1.18 | 0.91 | **0.99** | 0.84 | - | 1.16 | 0.87 |
|  | 2014 | **1.02** | 0.86 | - | 1.20 | 0.85 | **1.02** | 0.86 | - | 1.22 | 0.79 |
|  | 2015 | **0.97** | 0.79 | - | 1.17 | 0.72 | **0.98** | 0.83 | - | 1.17 | 0.83 |
|  | 2016 | **-** |  | - |  | - | **-** |  | - |  | - |
|  | 2017 | **0.78** | 0.64 | - | 0.95 | 0.01 | **0.79** | 0.65 | - | 0.94 | 0.01 |
|  | 2018 | **0.77** | 0.65 | - | 0.92 | 0.01 | **0.78** | 0.66 | - | 0.93 | 0.01 |
| **Gender** | Female | **-** |  | - |  | - | **-** |  | - |  | - |
|  | Male | **1.66** | 1.49 | - | 1.85 | 0.00 | **1.68** | 1.53 | - | 1.86 | 0.00 |
| **Age (years)** | <35 | **-** |  | - |  | - | **-** |  | - |  | - |
|  | >=35 | **0.95** | 0.86 |  | 1.06 | 0.36 | **0.96** | 0.86 |  | 1.06 | 0.41 |
| **Region** | East of England | **-** |  | - |  | - | **-** |  | - |  | - |
|  | London | **1.22** | 0.95 | - | 1.56 | 0.11 | **1.22** | 0.99 | - | 1.50 | 0.06 |
|  | South East | **1.08** | 0.84 | - | 1.37 | 0.55 | **1.07** | 0.86 | - | 1.33 | 0.53 |
|  | South West | **1.00** | 0.78 | - | 1.27 | 1.00 | **1.00** | 0.80 | - | 1.24 | 0.97 |
|  | West Midlands | **1.09** | 0.84 | - | 1.41 | 0.53 | **1.11** | 0.88 | - | 1.39 | 0.40 |
|  | North West | **1.10** | 0.86 | - | 1.40 | 0.46 | **1.09** | 0.89 | - | 1.33 | 0.41 |
| Yorkshire & Humber | | **1.28** | 1.00 | - | 1.65 | 0.05 | **1.29** | 1.04 | - | 1.60 | 0.02 |
|  | East Midlands | **0.90** | 0.70 | - | 1.16 | 0.41 | **0.89** | 0.71 | - | 1.10 | 0.28 |
|  | North East | **0.73** | 0.55 | - | 0.97 | 0.03 | **0.72** | 0.56 | - | 0.92 | 0.01 |
| ***Behavioural*** |  |  |  |  |  |  |  |  |  |  |  |
| **Injected drugs in the past year** | No | - |  | - |  | - | - |  | - |  | - |
|  | Yes | **1.26** | 1.12 | - | 1.41 | 0.00 | **1.26** | 1.13 | - | 1.41 | 0.00 |
| **Ever been in prison** | No | **-** |  | - |  | - | **-** |  | - |  | - |
|  | Yes | **1.16** | 1.04 | - | 1.29 | 0.01 | **1.14** | 1.01 | - | 1.29 | 0.03 |
| **Ever been homeless** | No | **-** |  | - |  | - | **-** |  | - |  | - |
|  | Yes | **1.15** | 1.02 | - | 1.29 | 0.03 | **1.17** | 1.04 | - | 1.31 | 0.01 |

## Appendix table 3

**Sensitivity analysis; odds of chronic HCV infection among antibody-positive PWID in England- comparing main model (samples missing HCV RNA imputed) with alternate model (samples missing HCV RNA excluded).**

|  |  | **Non-imputed model** | | | | | **Imputed (main) model** | | | | |
| --- | --- | --- | --- | --- | --- | --- | --- | --- | --- | --- | --- |
|  |  |  |  |  |  |  |  |  |  |  |  |
| **Variable** |  | **aOR** | **95% CI** | | | **p-value** | **aOR** | **95% CI** | | | **p-value** |
| ***Demographic*** |  |  |  |  |  |  |  |  |  |  |  |
| **Year** | 2011 | **1.02** | 0.84 | - | 1.23 | 0.83 | **1.04** | 0.87 | - | 1.25 | 0.80 |
|  | 2012 | **0.98** | 0.82 | - | 1.17 | 0.86 | **1.04** | 0.87 | - | 1.24 | 0.66 |
|  | 2013 | **0.93** | 0.78 | - | 1.11 | 0.43 | **0.99** | 0.84 | - | 1.16 | 0.87 |
|  | 2014 | **1.00** | 0.84 | - | 1.20 | 0.96 | **1.02** | 0.86 | - | 1.22 | 0.79 |
|  | 2015 | **0.91** | 0.76 | - | 1.09 | 0.30 | **0.98** | 0.83 | - | 1.17 | 0.83 |
|  | 2016 | **-** |  | - |  | - | **-** |  | - |  | - |
|  | 2017 | **0.72** | 0.60 | - | 0.87 | 0.00 | **0.79** | 0.65 | - | 0.94 | 0.01 |
|  | 2018 | **0.74** | 0.62 | - | 0.88 | 0.00 | **0.78** | 0.66 | - | 0.93 | 0.01 |
| **Gender** | Female | **-** |  | - |  | - | **-** |  | - |  | - |
|  | Male | **1.73** | 1.56 | - | 1.92 | 0.00 | **1.68** | 1.53 | - | 1.86 | 0.00 |
| **Age (years)** | <35 | **-** |  | - |  | - | **-** |  | - |  | - |
|  | >=35 | **0.94** | 0.85 |  | 1.05 | 0.26 | **0.96** | 0.86 |  | 1.06 | 0.41 |
| **Region** | East of England | **-** |  | - |  | - | **-** |  | - |  | - |
|  | London | **1.25** | 1.00 | - | 1.56 | 0.05 | **1.22** | 0.99 | - | 1.50 | 0.06 |
|  | South East | **1.05** | 0.83 | - | 1.31 | 0.70 | **1.07** | 0.86 | - | 1.33 | 0.53 |
|  | South West | **0.99** | 0.79 | - | 1.25 | 0.96 | **1.00** | 0.80 | - | 1.24 | 0.97 |
|  | West Midlands | **1.13** | 0.88 | - | 1.44 | 0.34 | **1.11** | 0.88 | - | 1.39 | 0.40 |
|  | North West | **1.12** | 0.90 | - | 1.39 | 0.31 | **1.09** | 0.89 | - | 1.33 | 0.41 |
| Yorkshire & Humber | | **1.31** | 1.04 | - | 1.65 | 0.02 | **1.29** | 1.04 | - | 1.60 | 0.02 |
|  | East Midlands | **0.91** | 0.72 | - | 1.16 | 0.46 | **0.89** | 0.71 | - | 1.10 | 0.28 |
|  | North East | **0.74** | 0.57 | - | 0.96 | 0.03 | **0.72** | 0.56 | - | 0.92 | 0.01 |
| ***Behavioural*** |  |  |  |  |  |  |  |  |  |  |  |
| **Injected drugs in the past year** | No |  |  | - |  |  | - |  | - |  | - |
|  | Yes | **1.27** | 1.14 | - | 1.42 | 0.00 | **1.26** | 1.13 | - | 1.41 | 0.00 |
| **Ever been in prison** | No |  |  | - |  |  | **-** |  | - |  | - |
|  | Yes | **1.14** | 1.01 | - | 1.27 | 0.03 | **1.14** | 1.01 | - | 1.29 | 0.03 |
| **Ever been homeless** | No |  |  | - |  |  | **-** |  | - |  | - |
|  | Yes | **1.17** | 1.04 | - | 1.31 | 0.01 | **1.17** | 1.04 | - | 1.31 | 0.01 |
